# Blood meal origins and insecticide susceptibility of *Anopheles arabiensis* from Chano in South-West Ethiopia

**DOI:** 10.1186/1756-3305-6-44

**Published:** 2013-02-22

**Authors:** Fekadu Massebo, Meshesha Balkew, Teshome Gebre-Michael, Bernt Lindtjørn

**Affiliations:** 1 Department of Biology, Arba Minch University, Arba Minch, Ethiopia; 2 Aklilu Lemma Institute of Pathobiology, Addis Ababa University, Addis Ababa, Ethiopia; 3 Centre for International Health, University of Bergen, Bergen, Norway

**Keywords:** *Anopheles arabiensis*, Human blood index, Bovine blood index, Pyrethroid insecticides, DDT, Insecticide resistance, South-West Ethiopia

## Abstract

**Background:**

*Anopheles arabiensis*, the main malaria vector in Ethiopia, shows both anthropophilic and zoophilic behaviours. Insecticide resistance is increasing, and alternative methods of vector control are needed. The objectives of this study were to determine the blood meal origins and the susceptibility to insecticides of *An*. *arabiensis* from Chano village near Arba Minch in South-West Ethiopia.

**Methods:**

Blood meal sources of anopheline mosquitoes collected using Centers for Disease Control and Prevention (CDC) light traps and pyrethrum spray catches (PSC) from human dwellings, and hand-held mouth aspirators from outdoor pit shelters were analysed using a direct enzyme-linked-immunosorbent assay (ELISA). The susceptibility of *An*. *arabiensis* to pyrethroid insecticides (alphacypermethrin, lambdacyhalothrin, deltamethrin, and cyfluthrin) and DDT was assessed using females reared from larval and pupal collections from natural breeding sites.

**Results:**

The blood meal origins of 2967 freshly fed *Anopheles* mosquitoes were determined. *An. arabiensis* was the predominant species (75%), and it fed mainly on cattle. The densities of both freshly fed *An. arabiensis* and those fed on human blood followed similar seasonal patterns. The overall human blood index (HBI) of *An. arabiensis*, including mixed blood meals, was 44% and the bovine blood index (BBI) was 69%. The HBI of *An. arabiensis* from CDC light trap collections was 75% and this was higher than those for PSC (38%) and outdoor pit shelter collections (13%), while the BBI was 65% for PSC, 68% for outdoor pit shelters and 72% for CDC light traps. More freshly fed and human blood-fed *An. arabiensis* were sampled from houses close to the shore of Lake Abaya (the major breeding site).

A high proportion of *An. arabiensis* was resistant to the pyrethroid insecticides, with a mortality rate of 56% for lambdacyhalothrin, 50% for cyfluthrin and alphacypermethrin, 47% for deltamethrin, and 10% for DDT.

**Conclusion:**

*Anopheles arabiensis* is the predominant species of anopheline mosquito in this region, and cattle are the main source of its blood meals. The greater tendency of this species to feed on cattle justifies the application of insecticides on cattle to control it. However, *An. arabiensis* has already developed resistance to the available pyrethroid insecticides, and alternative insecticides are needed for malaria vector control.

## Background

Malaria vectors that feed mainly on humans seriously affect human health because this behaviour increases the risk of malaria transmission [1]. The feeding pattern of *An. arabiensis,* the main vector of malaria in Ethiopia*,* varies among households [2]; it shows both zoophilic [3] and anthropophilic behaviours [4,5]. In Ethiopia, only a few studies have examined the blood meal origins of *An. Arabiensis*, particularly focusing on mosquitoes from animal sheds and human dwellings in the main malaria transmission seasons [3,5,6]. Such studies might have underestimated or overestimated the human–vector contact and the risk of malaria transmission [7].

Pyrethroid insecticides are widely used for bed net treatment, and for indoor residual spraying (IRS) [8] to reduce malaria incidence [9,10]. Long lasting insecticide treated nets (LLINs) and IRS have contributed to a reduction of malaria incidence in many malaria endemic countries by reducing the number of mosquitoes inside houses [11,12]. IRS and LLINs are efficient malaria vector control measures for *An. gambiae* s.s, which mostly feeds and rests indoors [13,14]. In contrast, *An. arabiensis* obtains a large proportion of its blood meals from cattle, apart from humans, and exhibits significant exophilic behaviour [4,6,13]. Thus, treatment of cattle with insecticide may reduce *An. arabiensis* populations in an alternative approach to malaria vector control [15,16]. In southern Ethiopia, Habtewold *et al.*[3] observed normal feeding behaviour of *An. arabiensis* on insecticide treated cattle with no diversion to humans. Moreover, Rowland *et al*. [15] reported a 56% reduction in the incidence of malaria in Pakistan resulting from the application of deltamethrin insecticide to cattle. In Africa, deltamethrin treated cattle provided protection against *An. arabiensis* in experimental huts [16].

Resistance to deltamethrin and permethrin in *An. arabiensis* has been reported from different parts of the country [17,18]. DDT resistant *An. arabiensis* is widespread in the country, including Arba Minch [17-19]. There has not been any information regarding susceptibility/resistance of *An. arabiensis* to pyrethroids from the area. Therefore, it is important to examine the insecticide susceptibility and blood meal origins of *An. arabiensis* from Chano in South-West Ethiopia for planning alternative or additional vector control approach.

## Methods

### Study area

The study was conducted in Chano, a village 15 km north of Arba Minch town in South-West Ethiopia, from May 2009 to April 2010. The village is located at 6°6.666′ N and 37°35.775^′^ E and at altitude of 1,206 m above sea level. There are three sub-villages, named sub-villages 1, 2 and 3. The village is close to Lake Abaya and sub-village 3 is found at a distance of 1350 to 1850 m from the lake. Three major irrigation canals pass through the village. The canals are permanent, well-constructed and flow into the agricultural fields outside the village. The inhabitants are subsistence farmers with maize cultivation and cattle ranching as their main source of income. The main cash crops are mangoes and bananas.

Domestic animals are usually kept in compounds in open conditions, but a few households use separate roofed cow shelters. It is not customary to keep animals in human dwellings. The people habitually sleep indoors throughout the year. There is no permanent or seasonal movement of animals out of the village for feeding or watering. The human population size is 6661 while the cattle population is 2217 (approximately three humans per head of cattle) (Table 1).

**Table 1:** Abundance of human and other potential blood meal hosts in the three sub-villages from Chano in Southwest Ethiopia

	**Human and other potential hosts**					
**Sub - villages**	**Human**	**Cattle**	**Goat**	**Sheep**	**Donkey**	**Chicken**
01	2289	568	90	112	36	261
02	2154	696	80	161	31	373
03	2218	953	83	166	42	557
Total	6661	2217	253	439	109	1191

The climate is hot and humid. Potential mosquito breeding sites are located at the shores of Lake Abaya and Harrae River. Small water bodies created by hoof-prints of cattle and hippopotami are the major breeding sites for *Anopheles* mosquitoes. Harrae River is a potential location for the breeding of anopheline mosquitoes during the dry seasons when many small water pouches are available. However, its influence is much smaller than that of Lake Abaya because it is about 5 km from the village. Monthly rainfall was recorded from the weather station in Arba Minch University, about 6 km from the study area, which is located at an altitude of 1200 m above sea level (the same as Chano village). In 2009, the annual rainfall was 645 mm, and in 2010 it was 1061 mm. The average minimum and maximum annual temperatures in 2009 were 17.8 and 32.2°C, and in 2010 they were 17.9 and 30.2°C.

### Study components and vector control activities in the area

This study is a part of the research programme “Ethiopian Malaria Prediction System,” which researches malaria and climate. The village was purposely selected, because it is one of the malarious villages in the Arba Minch area, for study of the epidemiological and entomological components of the disease and for the development of mathematical models to predict malaria. A recent publication by Loha and Lindtjørn described the occurrence of falciparum malaria in the village [20]. Antivector interventions, such as the application of IRS with DDT and distribution of insecticide treated nets (ITNs), were carried out by the government in June 2009 and March 2010, respectively. At least two bed nets were provided for each household.

### Mosquito collections

Mosquito sampling was conducted biweekly for a total of 12 consecutive months (May 2009 to April 2010) after obtaining verbal consent from the heads of households. Indoor blood-searching *Anopheles* were collected from ten randomly selected houses using Centers for Disease Control and Prevention (CDC) light traps (New Standard Miniature Light Traps 512 6 V 150A; John W. Hock, Gainesville, FL) by positioning the traps 45 cm above the floor at the feet of sleeping persons, who were protected by mosquito nets untreated with insecticide, from 18:30 to 6:00 hours [21]. Indoor resting mosquitoes were sampled in the mornings (6:00 to 9:00) from ten other randomly selected houses by application of the pyrethrum spray catch (PSC) method. Prior to spraying with an aerosol (Roach killer, M/S Kafr EI Zayat, Egypt with Registration No. ET/HHP/130) in each house, all food items and small animals were removed, the openings and eaves of windows and doors were filled with pieces of cloth, and the floor and furniture were covered with white sheets. Two sprayers, one from outside and the other inside the house were engaged, and knocked down mosquitoes were collected after ten minutes [22]. Outdoor resting mosquitoes were collected using a handheld mouth aspirator, paper cup and torch from ten pit shelters constructed under the shade of mango trees in the compound of ten randomly selected houses. Each shelter was 1.5 m deep and had an opening of 1.2 m × 1.2 m. About 0.5 m from the bottom of each pit shelter, a 30 cm horizontally deep cavity was prepared for each of the four sides [23]. The mouth of each pit shelter was covered with untreated bed net during collection periods (6:30–10:00 hours) to prevent mosquitoes from escaping.

### Mosquito processing

Live female anopheline mosquitoes were killed by freezing and all females were identified to species level using morphological characteristics [24]. Female anopheline mosquitoes were examined under a dissecting microscope and classified on the basis of their abdominal condition as unfed, freshly fed, half-gravid and gravid [22]. All female mosquitoes were preserved individually in vials with silica gel desiccant for later analysis (blood meal origins, parity rate and sporozoite rate).

### Detection of blood meal sources

The blood meal origins of freshly fed anopheline mosquitoes collected outside and inside houses were determined using a direct enzyme-linked immunosorbent assay (ELISA) following the method of Beier *et al.*[25] using human and bovine antibodies. Each mosquito abdomen was crushed in 50 μl phosphate buffered saline (PBS) solution (pH 7.4), which was further diluted by adding 950 μl PBS. Fifty microlitres of sample was added to each well in a 96-well microtitre plate, and incubated overnight at room temperature. Each well was washed twice with PBS containing Tween-20 solution, and 50 μl host specific conjugate (either human or bovine) was added to each well and incubated for one hour. After one hour, each well was washed three times with a PBS–Tween-20 solution. Finally, 100 μl of peroxidase substrate was added to each well and after 30 minutes the absorbance at 405 nm was recorded with an ELISA plate reader. Each blood meal sample was considered positive if the absorbance value exceeded the mean plus three times the standard deviation of the four negative controls (from a laboratory colony of *An. arabiensis* adults not fed with blood). Positive controls contained human and bovine blood.

### Species identification

Species specific polymerase chain reaction (PCR) [26] was carried out on 300 morphologically identified individuals from the *An. gambiae* complex obtained by random sampling for each month.

### Collection of aquatic forms and rearing to adulthood for susceptibility tests

*Anopheles* larvae and pupae were collected from natural breeding sites on the shores of Lake Abaya and along the Harrae River. They were reared to adulthood in the entomology laboratory at Arba Minch University in cages and provided with sterilized 10% sucrose solution soaked in cotton pads until testing. Before the test, *Anopheles* mosquitoes were identified using morphological keys [24] and those identified as from the *An. gambiae* complex (presumably *An. arabiensis*) were used for the test.

### Insecticide susceptibility tests

Insecticide susceptibility tests were carried out following the standard World Health Organization (WHO) protocol, using insecticide susceptibility test kits and insecticide-impregnated papers [27]. For each replicate, twenty non-blood-fed female *An. Arabiensis*, three to four days old, were exposed to papers impregnated with cyfluthrin (0.15%), lambdacyhalothrin (0.05%), alphacypermethrin (0.05%), deltamethrin (0.05%), and DDT (4%) for an hour. Controls were exposed to insecticide-free papers. The knockdown effect of each insecticide was recorded every five minutes during the one-hour exposure period [27]. Mosquitoes were then transferred to a recovery tube, supplied with sterilized 10% sucrose solution and kept in an insecticide free box for 24 hours, after which mortality rates were recorded. All susceptibility tests were carried out in a room with temperatures of 26.2–27.4°C and relative humidity of 72–84%. Four replicates of the tests and two replicates of the controls were carried out for each insecticide. For each replicate, new insecticide-impregnated paper was used.

### Data analysis

Data were entered and analysed using SPSS version 16 (SPSS Inc., Chicago. IL). The human blood index (HBI) and bovine blood index (BBI) were calculated as the proportion of the mosquitoes fed on either human or bovine blood meals out of the total blood meals determined [7]. Mixed (human + bovine) blood meals were added to the number of human and bovine blood meals when calculating the HBI and BBI [14,28]. Cryptic mixed blood meals were not analysed. The chi-squared test was used to compare the HBI and BBI of indoor and outdoor collected *An. arabiensis*. Analysis of variance (ANOVA) was used to compare the mean differences in the number of freshly fed *An. arabiensis* among months and sub-villages. The Tukey Honestly Significant Difference (HSD) test was used to distinguish the months with the maximum density of mosquitoes.

The results of the susceptibility tests were evaluated as recommended by WHO [27]. Mean mortality was determined across all batches of mosquitoes for a particular insecticide. Probit analysis was used to calculate KDT_50_ and KDT_90_ (the time taken to knock down 50% and 90% of mosquitoes, respectively).

## Results

### *Anopheles* species analysed for determination of blood meal origin

Overall, 3027 anopheline mosquitoes engorged with fresh blood were collected from May 2009 to April 2010, and 98% (n = 2967) of these were analysed to identify their blood meal origin. Of the 300 *An. gambiae* complex tested for speciation, 99.3% (n = 298) were *An. arabiensis* and two specimens did not amplify using PCR, and hence, their identity was unknown. Therefore, *An. arabiensis* was regarded as the only member of the complex and the predominant species (75%), followed by *An. marshalli* (22%) and *An. garnhami* (1.7%). *An. funestus*, *An. pharoensis* and *An. tenebrosus* accounted for 0.9%.

Seventy nine per cent of all *Anopheles* species, and 78% of *An. arabiensis*, gave positive reactions against human, bovine or both antibodies. Of all *Anopheles* mosquitoes analysed, 33.5% were found positive for mixed (human and bovine) blood meals. The host blood meals of 21% freshly fed *Anopheles* mosquitoes were not identified, and of these 57% (n = 360) were from outdoor pit shelters (Table 2).

**Table 2:** **Sources of blood meal of *Anopheles *mosquitoes collected indoors and outdoors from Chano in Southwest Ethiopia from May 2009-April 2010**

***Anopheles *spp.**		**Blood meals sources**			
	**No. analysed (HBI,%)**	**Human N (%)**	**Bovine N (%)**	**Mixed N (%)**	**Unknown N (%)**
*An. arabiensis*	2234 (44)	180 (8)	745 (33)	807 (36)	502 (22.5)
*An. marshalli*	656 (37)	68 (10)	308 (47)	175 (27)	105 (16)
*An. garnhami*	49 (37)	9 (18)	23 (47)	9 (18)	8 (16)
*An. funestus* group	16 (19)	0 (0.0)	6 (37.5)	3 (19)	7 (44)
*An. pharoensis*	7 (43)	1 (14)	0 (0.0)	2 (29)	4 (57)
*An. tenebrosus*	5 (20)	1(20)	1 (20)	0 (0.0)	3 (60)
Total	2967 (42)*	259 (9)	1083 (36.5)	996 (33.5)	629 (21)

Mixed (human + bovine) blood meals were added to the number of human and bovine blood meals when calculating the HBI; numbers in parenthesis. Unknown blood meals are negative for human and bovine antibodies. * Overall HBI of *Anopheles* mosquitoes.

*An. arabiensis* was the predominant species in outdoor pit shelters (64.8%), in space spray catches (84.6%), and in indoor CDC light traps (84.4%). *An. marshalli* (n = 436, 66.5%), *An. garnhami* (n = 35, 71.4%) and *An. funestus* group (n = 14, 88%) were caught more frequently in outdoor pit shelters, whereas *An. pharoensis* (n = 7) was caught only by indoor CDC light traps.

### Feeding behaviour of *Anopheles* mosquitoes

Table 2 shows the blood meal origins of *Anopheles* mosquitoes. *An. arabiensis* showed an overall preference for bovine bloods (33%) above human blood meals. Only 8% of *An. arabiensis* had obtained a blood meal from humans alone. The proportion of mixed blood meals (human–bovine) was high for *An. arabiensis* (36%). A large proportion of *An. arabiensis* had blood meals of unknown origin (22.5%). A high proportion of *An. arabiensis* from CDC light traps (65%) had blood meals of mixed origin, whereas the lowest proportion of mixed blood meals was obtained from outdoor pit shelters (10%). Few *An. arabiensis* from outdoor pit shelters (3%) had human blood meals alone. Similarly, *An. marshalli*, *An. garnhami*, and *An. funestus* group have shown a preference for bovine blood meals above human blood meals, with bovine blood meals alone in 47%, 47%, and 37.5% respectively. No *An. funestus* group had a human blood meal alone.

### Blood meal indices of *An. arabiensis*

Table 3 shows the blood meal origins of *An. arabiensis* from different collection sites. The overall human blood index (HBI) of *An. arabiensis*, including mixed blood meals, was 44%, while the bovine blood index (BBI) was 69%. The frequency of human–vector contact was much higher for mosquitoes caught in indoor CDC light traps than for indoor or outdoor resting samples collected by space spraying and from pit shelters. The proportion of human blood meals in *An. arabiensis* from indoor CDC light traps (75%) was significantly higher than for outdoor pit shelters (13%, χ^2^ = 288.7, p <.0001) and indoor resting space spray catches (38%, χ ^2^ = 36.6, p <.0001). Indoor resting *An. arabiensis* had a HBI of 38% which was significantly higher than the 13% obtained for samples from outdoor pit shelters (χ ^2^ = 58.8, p <.0001). The proportion of bovine blood meals in *An. arabiensis* was similar for indoor resting (65%), outdoor pit shelter resting (68%) and CDC light trap (72%) samples.

**Table 3:** **Blood meal origins of *Anopheles arabiensis *collected indoors and outdoors from Chano in Southwest Ethiopia**

		**Blood meal origins**			
**Collection sites**	**No. analysed (HBI,%)**	**Human N (%)**	**Bovine N (%)**	**Mixed N (%)**	**Unknown N (%)**
Indoor CDC light traps	988 (75)	94 (9.5)	70 (7)	644 (65)	180 (18)
Space sprays catches	352 (38)	59 (17)	154 (44)	74 (21)	65 (18.5)
Outdoor pit shelters	894 (13)	27 (3)	521 (58)	89 (10)	257 (29)
Total	2234 (44)*	180 (8)	745 (33)	807 (36)	502 (22.5)

Mixed (human + bovine) blood meals were added to the number of human and bovine blood meals when calculating the HBI; numbers in parenthesis. Unknown blood meals are negative for human and bovine antibodies. * Overall HBI of *An. arabiensis.*

### Household and seasonal variations in density of blood fed *An. arabiensis*

**Figure 1:**
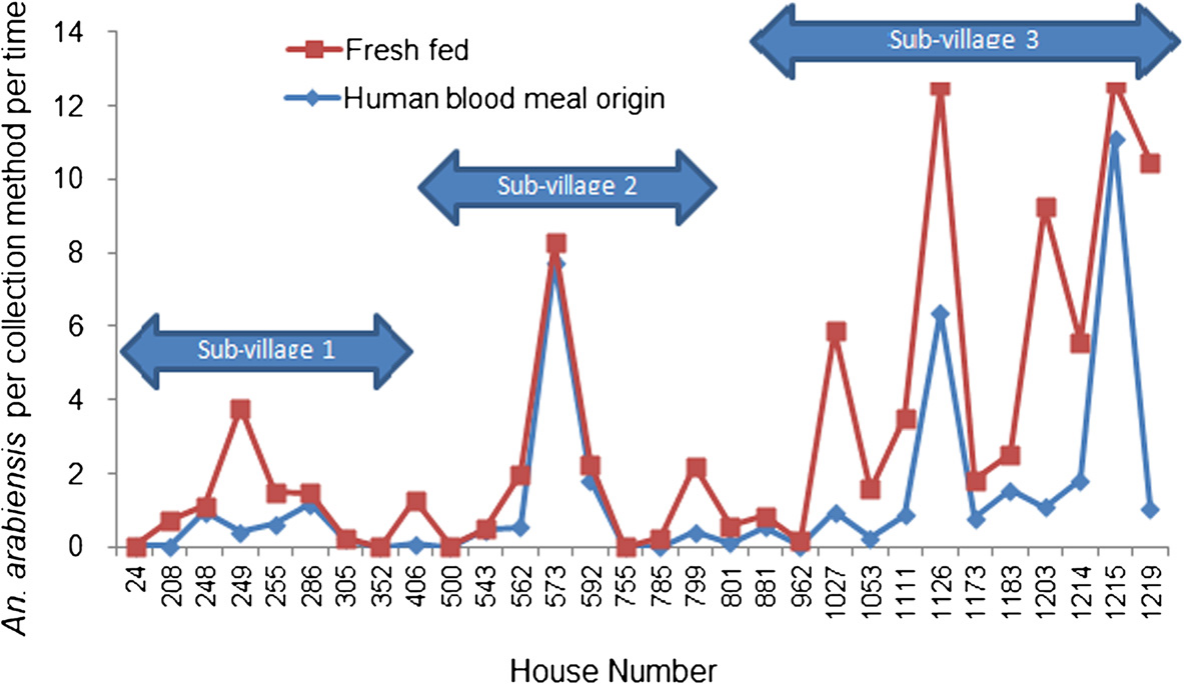
**Variation of fresh fed and human blood fed *An. arabiensis *among the three sub-villages from Chano in southwest Ethiopia. (Pyrethrum spray catches:** 24, 255, 352, 543, 785, 801, 962, 1111, 1183 & 1214; **CDC light traps:** 248, 286, 305, 573, 592, 755, 881, 1126, 1173 & 1215; **Pit shelter:** 208, 249, 406, 500, 562, 799, 1027, 1053, 1203 & 1219).

The densities of freshly fed *An. arabiensis* varied significantly among the three sub-villages (F = 5.0; df = 2; p = 0.02). Figure 1 shows the variations in freshly fed and human-blood-fed *An. arabiensis* among the three sub-villages. The maximum number of freshly fed *An. arabiensis* was collected in houses in the sub-village nearest to the major breeding site (between 1350 m and 1850 m), with 12.6 per CDC light trap per night, 10.5 per pit shelter and 6 per hut PSC. In contrast, in sub-village 2 (located between 1960 m and 2270 m from the major breeding site), the maximum number of freshly fed *An. arabiensis* was 8.3 per CDC light trap per night, 2.2 per pit shelter per collection time and 0.6 per hut PSC. The maximum number of freshly fed *An. arabiensis* was 1.5 per CDC light trap per night, 4 per pit shelter and 1.5 per hut PSC in sub-village 1 (located between 2350 m and 2600 m from the major breeding site). Similarly, the number of human*-*blood-fed *An. arabiensis* was highest in sub-village 3, with 11 fed on human blood per CDC trap per night, 1.8 human fed per hut PSC and 1 human fed per pit shelter.

**Figure 2:**
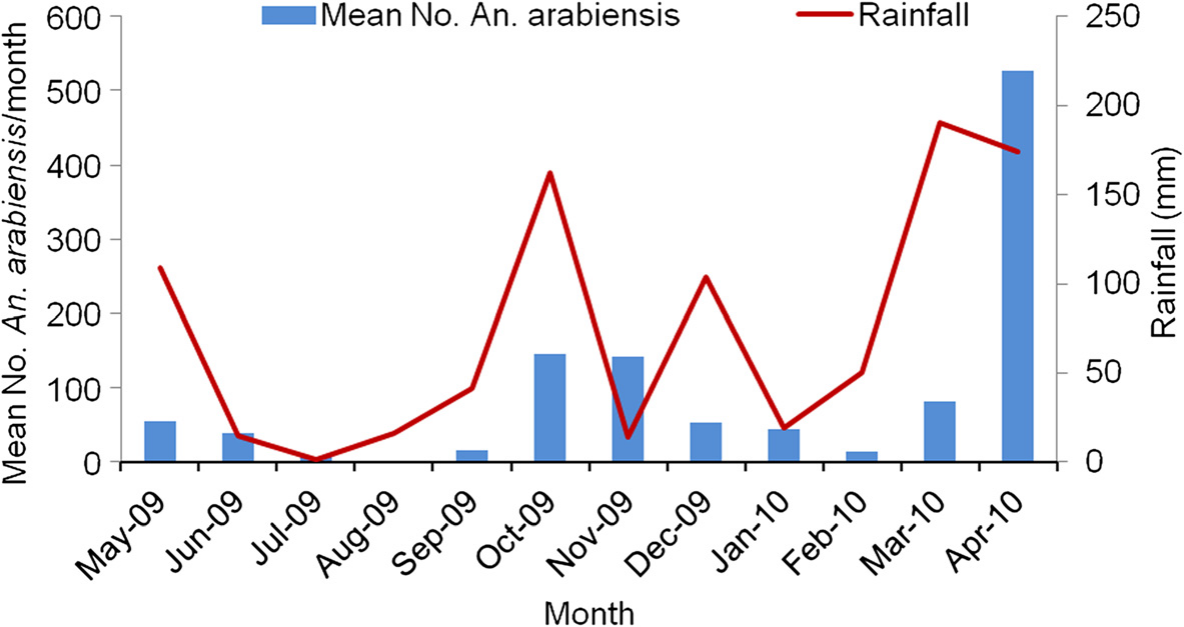
**Monthly rainfall (in mm) and the mean density of fresh fed *Anopheles arabiensis *from Chano in southwest Ethiopia.**

**Figure 3:**
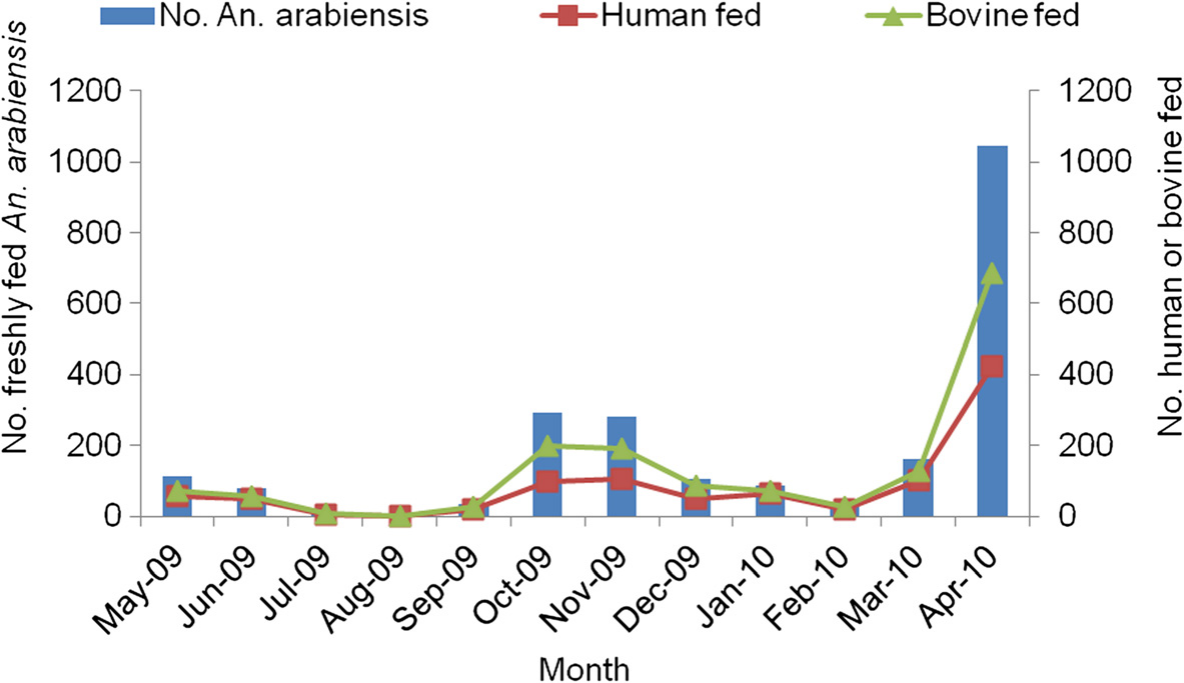
**Number of freshly fed, human and bovine blood fed (mixed blood meal included in both human and bovine) *Anopheles arabiensis *from Chano in southwest Ethiopia.**

The density of *An. arabiensis* varied with season (F = 3.67; df = 11; p = 0.017) and was associated with rainfall (Figure 2). The density of the total number of freshly fed, human and bovine blood engorged *An. arabiensis* followed a similar seasonal pattern (Figure 3). The highest number of freshly fed *An. arabiensis* was collected in April 2010, comprising 24.7 mosquitoes per CDC light trap, 20.8 mosquitoes per pit shelter and 6.9 mosquitoes per hut in space spray catches. In April 2010, we collected the highest number of *An. arabiensis* with meals of human blood origin from indoor CDC light traps: 16.3 human-blood-fed per CDC light trap per night, 2.5 human-blood fed per pit shelter and 2.4 human-blood fed per PSC. The number of freshly fed *An. arabiensis* declined to zero in August 2009, following the period of lowest rainfall in the preceding two months. The highest densities of *An. arabiensis* were collected during October and November 2009, and in April 2010. However, significantly higher densities of freshly fed *An. arabiensis* were collected in April 2010 than in October and November 2009 (Tukey HSD test, p= 0.004).

### Knockdown and mortality of *An. arabiensis*

Table 4 shows the knockdown time for the five insecticides used with *An. arabiensis*. Only deltamethrin resulted in 100% knockdown, with the lowest KDT_50_ (21 minutes) and KDT_90_ (35 minutes) values, whereas DDT resulted in only 10% knockdown within 60 minutes of exposure time. The KDT_50_ values of alphacypermethrin, cyfluthrin and deltamethrin were 27, 25 and 21 minutes, respectively. Only cyfluthrin and deltamethrin resulted in more than 90% knockdown within 60 minutes of exposure time. The KDT_50_ value of lambdacyhalothrin was 1.9 times, and that of alphacypermethrin was 1.3 times, higher than that of deltamethrin.

**Table 4:** **Percent knockdown, knockdown time (KDT) (in minutes) and mortality rates of *Anopheles arabiensis *exposed to pyrethroids and DDT from Chano in Southwest Ethiopia**

**Insecticides tested**	**Number exposed**	**% knockdown**	**KDT**_**50**_**(95% CI)**	**KDT**_**90**_**(95% CI)**	**% mortality (±SE)**	**Status**[27]**(<80%)**
Lambdacyhalothrin (0.05%)	80	80	39 (36–43)	**	56 ± 9.6	resistant
Alphacypermethrin (0.05%)	80	89	27 (20–32)	**	50 ± 5.4	resistant
Cyfluthrin (0.15%)	80	96	25 (19–29)	42 (37–51)	50 ± 9.5	resistant
Deltamethrin (0.05%)	80	100	21 (18–23)	35 (31–39)	47 ± 3.2	resistant
DDT (4%)	80	10	*	**	10 ± 3.5	resistant

* 50% was not knocked down ** 90% was not knocked down, CI= confidence interval, SE = standard error.

The mortality rates of *An. arabiensis* after the 24-hour recovery period was 56% for lambdacyhalothrin, 50% for cyfluthrin and alphacypermethrin, 47% for deltamethrin and only 10% for DDT, much lower than the susceptibility boundary of 80% (Table 4). The mortality rate calculated for the experimental tests was not corrected because mortality in the controls was always less than 5%.

## Discussion

The results of this study showed that *Anopheles arabiensis* is the predominant anopheline species in the area, and it feeds mainly on cattle. *An. arabiensis* has already developed resistance to the available pyrethroid insecticides and alternative insecticides may be needed for the treatment of cattle. Houses close to the main mosquito breeding site harboured more freshly fed *An. Arabiensis* and those fed on human blood*.*

Earlier studies from Ethiopia have examined the blood meal origins of *An. arabiensis* from animal sheds and human dwellings during the main malaria transmission seasons only [3,5,6], neglecting the dry months. A strength of our study is that the blood meal origins of freshly fed *An. arabiensis* were determined by collecting mosquitoes from outdoor pit shelters and inside houses throughout a year, as was recommended by Garrett-Jones [7]. Mosquitoes were sampled from 30 collection sites every two weeks each month and, hence, their blood meals are representative of human contact with the mosquito vector. Our data compare well with those of Loha and Lindtjørn [20], who studied the incidence of malaria in the same village and reported the highest incidence of malaria in the nearest village to Lake Abaya (sub-village 3), where we found the highest densities of freshly fed and human-fed *An. arabiensis*.

One limitation of our study was the inability to determine the cryptic mixed blood meals of malaria vectors that had fed on different individuals of the same species. This might have led to underestimation of human–vector contact and pathogen transmission intensity, as was reported by Norris *et al.*[29] and Scott and Takken [1]. Another limitation is that we could not identify other animal sources of blood meals for malaria vectors in addition to humans and cattle. Such information may be important in the planning of vector control options. The failure to determine the blood meal origins of some freshly fed *An. arabiensis* may have occurred because we lacked antibodies for other hosts, or it could have resulted from enzymatic degradation of the blood.

Many zoophilic *An. arabiensis* were collected indoors using space spray catches after they had fed on cattle outdoors, which provides clear evidence for preference of a bovine blood meal over human. The zoophilic behaviour of *An. arabiensis* observed in this study is consistent with other findings from Ethiopia [3,6]. The HBI (38%) of *An. arabiensis* from space spray catches was lower than the HBI from southern Zambia (92.3%) [30], the Kenyan coast (91%) [31], Konso in southern Ethiopia (55.2%) [3] and the Gambia (82%) [32], but higher than from Eritrea (20%) [33] and western Kenya (23%) [13]. The percentage of mixed blood meals for indoor resting *An. arabiensis* (21.0%) was comparable with that found in other studies [13,34]. No mixed blood meals were identified in resting *An. arabiensis* from inside houses in Kenya [31].

The *An. arabiensis* collected using CDC light traps had higher HBI than those from indoor resting and outdoor pit shelters. Fornadel *et al.*[35] reported an HBI of 94% for *An. arabiensis* from southern Zambia collected using CDC light traps. Interestingly, a high proportion of *An. arabiensis* from indoor CDC light traps had mixed blood meals (65.2%). This suggests that they were interrupted while feeding outdoors on cattle and moved into houses to complete their feeding in a single night or on consecutive nights [29,36]. The lowest HBI was found for *An. arabiensis* from pit shelters located near cattle that are kept outdoors. This reveals that the accessibility of hosts influences the feeding behaviour of this species, as also reported by others [37]. This is the first report of the HBI of *An. marshalli* and *An. garnhami*. Future studies should be conducted to examine the sporozoite rate of these species to determine their possible role in malaria transmission.

The few *An. funestus* collected from outdoor pit shelters was found with cattle blood meal. Unfortunately, we did not identify the species group using molecular method. However, the occurrence of some species from larval identification is known in Ethiopia [38]. Of the members of the group, *An. parensis* and *An. rivulorum* are regarded to be zoophilic elsewhere in Africa [39,40]. *An. funestus* has been incriminated as an anthropophilic and endophilic malaria vector in many countries in Africa [41]. Therefore, the *An. funestus* group identified morphologically in this study could be either *An. rivulorum* or *An. parensis* or both .

In this study area, the distribution of the malaria vector was seasonal. The maximum number of freshly fed and human blood meal-engorged *An. arabiensis* was recorded one month after the peak rainfall*.* Possible reasons are that the rainfall in the previous month may have provided more breeding sites and increased the relative humidity, which contributes to a high density and longevity of the vectors and consequently increases human–vector contact [42]. In particular, the longevity of the vector is crucial for disease transmission because it increases the chance of an infectious bite occurring [42]. Kristan *et al.*[43] have also shown a one month lag after rainfall as a predictor of vector density in the African highlands. A study from Eritrea also has shown an increase in the *An. arabiensis* population one month after the start of rainfall [33]. Moreover, the distribution of *An. arabiensis* was influenced mainly by the location of breeding sites on the shore of Lake Abaya. A study from the same area [20] and one from Northern Tanzania [44] showed a higher risk of malaria infection in a population living near to mosquito breeding sites. To locate and identify households at greater risk of malaria is, therefore, crucial in the planning and implementation of vector control approaches.

*An. arabiensis* showed a high level of resistance to knockdown and mortality in response to pyrethroid insecticides (deltamethrin, alphacypermethrin, lambdacyhalothrin and cyfluthrin) and DDT. The knockdown resistance was most likely due to the possession of various detoxifying enzymes. Studies from East and Central Africa [45,46] have reported the occurrence of high levels of mono-oxygenase enzymes in resistant *An. arabiensis*. Elevated levels of mixed function oxidases and β-esterases were also reported in resistant *An. arabiensis* in Tanzania [47]. Moreover, the West African *kdr* mutation (L1014F) detected in high frequencies in South-West and Northern Ethiopian *An. arabiensis* populations [18,48] could be another reason for high knockdown resistance in the study area. The KDT_50_ of lambdacyhalothrin (39 minutes) was higher than that of the other pyrethroid insecticides, but shorter than that reported from Senegal (43.6 minutes) in *An. gambiae*[49]. Compared with studies from Ethiopia, the KDT_50_ values of 25.3 minutes for *An. arabiensis* from Gorgora and 37.6 minutes from Ghibe were higher than that we observed for deltamethrin (21 minutes), but similar to that reported from Sodere (21.9 minutes) [18]. The impact of knockdown resistance is that it can allow the vectors to bite humans even inside the long lasting insecticide treated nets (LLINs) because the vector can withstand a long duration of exposure without being knocked down [50].

The high level of resistance of *An. arabiensis* to deltamethrin and DDT is not surprising because of the long history of the use of DDT for IRS and the widespread use of deltamethrin for LLINs and IRS, and cross-resistance may occur [51]. The high level of DDT (90%) resistance in *An. arabiensis* was expected because 60% resistance was reported from South-Western Ethiopia 14 years ago [19]. The mortality rate (10%) due to DDT was slightly higher than that reported by http://Yewhalaw and his colleagues [17,48] but lower than that observed by Balkew *et al.*[18,52]. The mortality rate due to deltamethrin (47%) was lower than that observed in other studies in Ethiopia [17,18,48].

The resistance of *An. arabiensis* to alphacypermethrin, lambdacyhalothrin and cyfluthrin was unexpected because they have not been used for vector control. This implies that the use of insecticides with similar modes of action could shorten the duration of efficacy of other insecticides of the same class once resistance has developed in the mosquito population [53]. The most likely explanation is the presence of cross-resistance between insecticides of the same group [51], which might limit the choice of alternative insecticides for vector control. Cross-resistance between DDT and permethrin has been reported in Ethiopia [18] in *An. arabiensis*. No information is available in Ethiopia about the resistance of *An. arabiensis* to alphacypermethrin, lambdacyhalothrin and cyfluthrin. A study from Ghana has shown high survival rates of *An. gambiae* s.s after exposure to cyfluthrin and lambdacyhalothrin [54].

The results obtained in this study have implications for vector control. *An. arabiensis* showed a tendency to feed more frequently on cattle than on humans. In similar settings, Mahande and colleagues [16] and Rowland *et al.*[15] reported the success of treatment of cattle with pyrethroid insecticides in controlling zoophilic malaria vectors. Moreover, the preference of *An. arabiensis* to rest indoors after feeding on cattle outdoors in an area that practises indoor-based vector control activities could explain the low efficacy of LLINs and IRS, owing to the resistance of *An. arabiensis* to pyrethroid insecticides. Previously, N’Guessan *et al.*[55] reported a low efficacy of LLINs and IRS in areas with resistant malaria vectors. On the other hand, the indoor resting preference of *An. arabiensis* is an opportunity to use current indoor based antivector strategies [56] because mosquitoes inside houses are easily targeted [57], but appropriate management of insecticide resistance needs to be implemented.

The possible explanation for the higher HBI and presence of mixed blood meals in *An. arabiensis* from indoor CDC light traps may be that most people are bitten indoors before they go to bed, or that protection from indoor antivector interventions is reduced by the presence of pyrethroid-resistant *An. arabiensis.* In the same setting, Loha and Lindtjørn [20] described the personal protection role of LLINs, with no impact on community members who did not use the nets. It is the killing capacity that provides protection from the infectious bites of malaria vectors for people in the community who do not use bed nets [58]. In an area with pyrethroid-resistant malaria vectors, even the combination of LLINs and IRS has a low impact on the prevalence of malaria [59], and in other settings an increase in malaria cases has been reported [60]. Asidi *et al.*[61] showed that the treatment of bed nets with pyrethroid insecticides provides additional protection from mosquito bites only if the vectors are susceptible to the chemicals. Our findings also show that the density of freshly fed and human blood-fed *An. arabiensis* increased in April 2010 despite the mass distribution of bed nets in March 2010. Hence, it is advisable to introduce additional vector control strategies that target a reduction in the entry of blood-searching vectors into houses and diversion to alternative hosts available outdoors. However, we should not underestimate the fact that malaria transmission can occur outdoors via human-biting mosquitoes, even if the HBI is low [62].

In addition, the finding of the lowest HBI and percentage of mixed blood meals in *An. arabiensis* from outdoor pit shelters suggests that *An. arabiensis* is less likely to leave houses after feeding indoors on humans [13], or that people are bitten outdoors less frequently in the area. Therefore, IRS and LLINs can provide successful protection from malaria infection if the vectors are susceptible to the available pyrethroid insecticides.

## Conclusion

Although a high propensity for *An. arabiensis* to feed on bovine blood was observed in our study area, treatment of cattle with insecticides may not reduce the vector density because *An. arabiensis* has already developed resistance to the available pyrethroid insecticides that are recommended for the treatment of cattle. Thus, alternative insecticides with different modes of action may be needed for treatment of cattle.

## Competing interests

The authors have no conflict of interest.

## Authors’ contributions

FM: Project design, conducted field and laboratory work, data analysis and interpretation, wrote the draft of manuscript, MB: Project design, field and laboratory supervision, and manuscript revision, TG: Project design, supervision and manuscript revision, BL: Project design, field supervision, provided statistical input and manuscript revision. All authors read and approved the final manuscript.
